# Insights into the Supramolecular Structure and Degradation Mechanisms of Starch from Different Botanical Sources as Affected by Extrusion-based 3D Printing

**DOI:** 10.1021/acs.biomac.2c00881

**Published:** 2022-12-02

**Authors:** Mahdiyar Shahbazi, Henry Jäger, Rammile Ettelaie, Marco Ulbrich

**Affiliations:** † Institute of Food Technology, University of Natural Resources and Life Sciences (BOKU), Muthgasse 18, 1190Vienna, Austria; ‡ Food Colloids Group, School of Food Science and Nutrition, University of Leeds, LeedsLS2 9JT, U.K.; § Department of Food Technology and Food Chemistry, Chair of Food Process Engineering, Technische Universität Berlin, Office GG2, Seestraße 13, D-13353Berlin, Germany

## Abstract

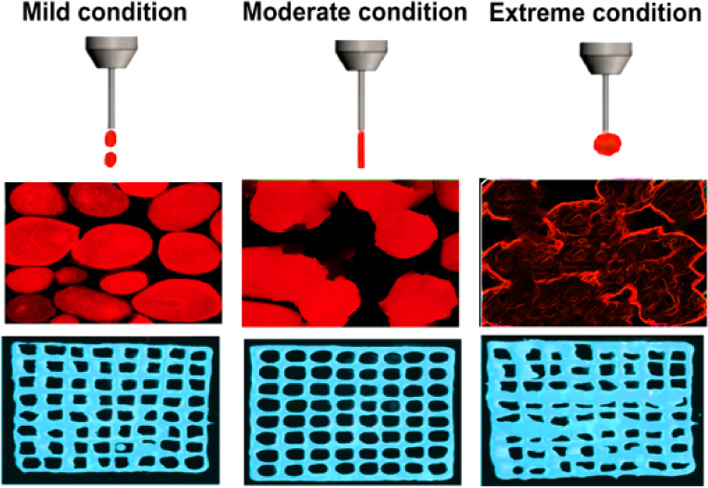

Extrusion-based 3D printing has emerged as the most versatile additive manufacturing technique for the printing of practically any material. However, 3D printing of functional materials often activates thermo-mechanical degradation, which affects the 3D shape quality. Herein, we describe the structural changes of eight different starch sources (normal or waxy) as a consequence of the temperature of an extrusion-based 3D printing system through in-depth characterization of their molecular and structural changes. The combination of size-exclusion chromatography, small-angle X-ray scattering, X-ray diffraction, dynamic viscoelasticity measurements, and *in vitro* digestion has offered an extensive picture of the structural and biological transformations of starch varieties. Depending on the 3D printing conditions, either gelatinization was attained (“moderate” condition) or single-amylose helix formation was induced (“extreme” condition). The stiff amylopectin crystallites in starch granules were more susceptible to thermo-mechanical degradation compared to flexible amorphous amylose. The crystalline morphology of the starch varieties varied from B-type crystallinity for the starch 3D printing at the “moderate” condition to a mixture of C- and V-type crystallinity regarding the “extreme” condition. The “extreme” condition reduced the viscoelasticity of 3D-printed starches but increased the starch digestibility rate/extent. In contrast, the “moderate” condition increased the viscoelastic moduli, decreasing the starch digestion rate/extent. This was more considerable mainly regarding the waxy starch varieties. Finally, normal starch varieties presented a well-defined shape fidelity, being able to form a stable structure, whereas waxy starches exhibited a non-well-defined structure and were not able to maintain their integrity after printing. The results of this research allow us to monitor the degradability of a variety of starch cultivars to create starch-based 3D structures, in which the local structure can be controlled based on the 3D printing parameters.

## 1 Introduction

Three-dimensional (3D) printing technology is a promising leading rapid prototyping process to manufacture highly intricate 3D objects. The extrusion-based printing technique has become one of the most used 3D printing technologies in the field of pharmaceutical and food research thanks to its inexpensive and small equipment, the possibility of drawing prototypes with a wide range of intricated geometries, and the non-use of organic solvents.^1^ The 3D printing through the material extrusion of viscoelastic inks is also known as direct-ink-writing (DIW) or robocasting, in which a delivery system (*i.e.*, an extruder) precisely deposits precise levels of viscoelastic and thixotropic polymers over variable distances. Compared to conventionally fabricated 3D structures, DIW-printed ones have more complex shapes, greater precision, enhanced productivity, and even improved performances that arise from well-designed architectures.^2−5^ It is apparent that the DIW parameters (pressure, speed, and nozzle size) and printing environments (temperature, direct writing medium) have a strong impact on the 3D printing process. Only proper parameters and environments can make the printable ink form a stable 3D structure.^6^

The processing of biopolymers through DIW printing commonly induces consistent mechanical and thermal stresses to the biomaterials. The high values of shear and heat to which the biopolymers are exposed upon 3D printing cause thermo-mechanical degradation.^7,8^ As a general rule, if the depolymerization favorably proceeds through chain cleavage, the molar mass distribution plot shifts toward the lower molar masses.^7^ The outcome is the reverse of the degradation performed by the crosslinking reaction.^7,9^ As a result, the physicomechanical and structural features of the materials are altered. The nature of these changes and the extent of polymer degradation are strongly related to the inherent features of the materials and the degradation environment.^10^

Starch has a wide range of applications in 3D printing due to its low cost and availability.^2,11^ As a biodegradable polymer, it is a promising resource for an innovative generation of biomaterials to replace some petroleum-based polymers. Accordingly, there is increasing attention to understanding the interaction relationship between the molecular and structural behavior properties of starches used for the 3D printing process. It has been reported that the molecular, crystalline, and mechanical features of 3D-printed starch-based constructs directly relate to the ratio of amylose (AM) and amylopectin (AP).^12^ The starches obtained from different cereal sources have different levels of AM and AP,^13^ which consequently can rationally affect printability and shape fidelity. During the 3D printing process, the starch granules are hydrated, swollen, and broken down, and the starch polysaccharides are partially depolymerized.^7^ Therefore, they are converted to a viscoelastic gel, accompanied by a change in the molecular or supramolecular level and rheological properties of starch. Since extrusion 3D printing contains high-shear and high-pressure stresses, starch gelatinization can be commonly processed at a low moisture content as the extrusion shearing force physically breaks down the granules, which offers quicker water transmission into the internal components. In this case, the reduced crystallinity does not result from the water penetration upon 3D printing but from the mechanical breakdown of the intermolecular linkages as a result of the severe shearing force upon the process.^14^ Reportedly, following the application of high shear at the low moisture content, minor extents of melted or gelatinized granules, along with granule disintegration, exist simultaneously.^7^

The main objectives of most starch processing techniques are melting and mixing, which are adjusted to minimize molecular degradation. Upon extrusion 3D printing, starch fragmentation is reasonably inevitable, which depends on 3D printing parameters including temperature, nozzle diameter, and moisture content, as well as the starch source. Once subjected to moderately elevated temperatures, starch shows depolymerization caused by chain cleavage and transfer reactions, comprising an initial quick reduction of molecular mass. It was reported that thermal degradation below gelatinization temperature is very restricted and often negligible.^7,14^ Thermal depolymerization of starch is also induced with a random chain cleavage, although after unzipping and development of low molar mass portions. According to the literature, there are at least five molecular levels for starch.^15^ The formation of linear branches of anhydroglucose units (AGU) by α-(1–4)-glycosidic bonds (level 1) linked together with α-(1–6)-glycosidic linkages as a branching point to develop a completely branched separate molecule (level 2), mostly AM and AP. AM is typically linear with a few long branches, whereas AP has a highly branched structure with branch points of about 5% and a high level of short branches. The clusters of double helices are formed by external parts of AP branches that build up the crystalline lamellae.^7^ On the other hand, the amorphous lamellae contain internal parts accompanied by branching points (typically called building blocks).^15^ The alternating amorphous and crystalline lamellae (level 3) with a repeat distance of about 10 nm^16^ together develop the semi-crystalline growth rings (level 4) within the starch granules (level 5).^17^ AM exists in either a single helical conformation or an amorphous state that is interspersed between AP components.^18^

Extrusion 3D printing is frequently applied for processing starch, offering a stable and semi-continuous process. However, starch printing is more complicated compared to synthetic polymers because of the complex structure of starch and molecular alterations upon 3D printing. Furthermore, the final functionality of starch is associated with more than one structural level, including molar mass and crystalline structure. Therefore, it is imperative to understand the multi-level structural changes during 3D printing to enhance the functional features of starch polymers. In a very recent study, we evaluated the phase transition of starch (such as the gelatinization mechanism) under the different temperatures of an extrusion-based 3D printing system.^7^ However, this simulation does not fully represent the starch multi-level structural changes during printing processing, where different contents of AM from starch botanical source varieties are commonly used during processing. Furthermore, it is not well understood how the thermal printing process affects starch structures at each level. Therefore, a thorough understanding of the decomposition of starch from different botanical sources is critical to evaluating the macromolecular and conformational properties of starch.

Considering this fact, there is no study reporting the changes in molecular properties of different starch types as affected by the 3D printing process. Herein, we aimed to assess the printing quality and molecular behavior of the 3D-printed starches based on eight starch varieties as influenced by the temperature of an extrusion-based 3D printer. Starches with different AM/AP ratios are of interest as these two polymer fractions act very uniquely from a technological viewpoint. The printing performance and molecular/supramolecular properties of 3D-printed structures can be strongly affected by the type of starch and therefore that of the AM/AP ratio. The thermo-mechanical degradation effects of DIW 3D printing under “mild”, “moderate”, and “extreme” temperatures on the molecular behavior of starch varieties were discussed profoundly.

## 2 Materials and Methods

### 2.1 Starch Varieties

The starch samples used and their respective sources were regular maize starch (RM), high-amylose maize starch (HAM), waxy maize starch (WM) (Sigma-Aldrich, Steinheim, Germany), regular potato (RP) and waxy potato (WP) starches (National Starch and Chemical Company, Bridgewater, NJ), regular wheat starch (RW), barley (RB), and native waxy barley (WB) starches (Grain Processing Corporation, Muscatine, IA), which are a selection of important starches for the pharmaceutical application and food industry. The investigations of chemical and physicochemical properties of starch varieties, such as AM/AP ratio, water-holding capacity (WHC), and damage starches, and the determination of lipid, protein, and water contents are included in the Supporting Information (Section S1). Dimethyl sulfoxide (DMSO) was purchased from Sigma-Aldrich (Steinheim, Germany). *para*-Hydroxybenzoic acid hydrazide assay was performed (H9882, Steinheim, Germany). Isoamylase from *Pseudomonas* sp. was obtained from Megazyme International, Bray, Co. (Wicklow, Ireland). Milli-Q water was used in all instances. All other chemicals were used as received without further purification.

### 2.2 Pre-preparation of Starch Sources for DIW 3D Printing

All the native starches were obtained as freeze-dried powders that were simply redispersed in water through the application of a shear force process. However, there was evidence of some clumps during the storage of starch powders with the attendance of the large aggregates. For this reason, a pre-treatment method was employed to develop the fine powders, which were used in the printing process.^7^ The native starches were completely dispersed in Milli-Q water (100 g L^–1^) at ambient temperature and gently stirred using a magnetic stirrer for 60 min. Then, the starch suspension was sheared using a high-speed rotor-stator device (Ultra-Turrax, IKA T25 digital, Staufen, Germany) at a shear rate of 56 s^–1^ for 10 min. After completing the process, the sheared samples were collected and dried in an oven at 40 °C for 36 h. Next, the dried samples were ground to disrupt the clumps/agglomerations and filtered using a sieve to attain a particle size of about 30 μm.

### 2.3 Extrusion-based 3D Printing Process

Before the 3D printing process, the tested starch was initially conditioned in a desiccator including a saturated potassium sulfate solution (relative humidity of 97%) at ambient temperature for 6 days to reach a moisture content of 44 g/100 g. For the printing process, three typical 3D models were designed and selected to further evaluate the printing fidelity of the starch ink. In this regard, computer-aided design software (AutoCAD; Autodesk Inc., San Rafael, CA) was used to model the lattice matrix, lattice square, and gradient spacing objects, which converted them to a stereolithography (.stl) file. To control XYZ direction instruction for the printers, a print path was obtained by the development of the G-code files, offered by the open-source CAM software Slic3r (slic3r.org, consulted on November 2021) from the STL (.stl) file. The extrusion printer was a micro-dispensing pump system (nScrypt-3D-450. nScrypt, Orlando, FL) equipped with a syringe pump (PHD Ultra; Harvard Apparatus, Holliston, MA). The printable starch-based inks were filled with a stainless-steel container (10 mL) and stirred with a Vortex mixer (Fisher Scientific, Ontario, Canada) for 5 min, therefore eliminating any air bubbles from the ink. The nozzle tip was elevated by 1 mm upon completion of the construction of each layer and before beginning the fabrication of the subsequent layer. The printing process was performed at three different processing conditions (“mild” 40% moisture content, 40 °C, and 0.50 mL min^–1^ extrusion flow speed; “moderate” 40% moisture content, 80 °C, and 0.50 mL min^–1^ extrusion flow speed; “extreme” 40% moisture content, 120 °C, and 0.50 mL min^–1^ extrusion flow speed). In addition to these conditions, the native starches were also printed with no heating-induced printing temperature (at the ambient conditions) in order to better compare the effect of printer temperatures. The process continued until a proper 3D structure was printed in each case. The number of deposited layers was 8, and the width of the tip was 1 mm.^7^ Each 3D-printed starch was immediately used for analytical and instrumental measurements to avoid retrogradation. For ease of understanding, different codes were used for the starch varieties and samples that had been printed at different printer temperatures. For example, the codes RW-40, RW-80, and RW-120 were considered for the RW starches that were 3D-printed at 40, 80, and 120 °C, respectively. The non-printed samples were the native starches with no printing process.

### 2.4 Dissolution and Debranching of Starch Polymers

To produce a reliable route for monitoring the molecular state of starch, the 3D-printed objects (5 mg) were dissolved in DMSO (2 mL) including lithium bromide (0.5 wt %), which was vigorously stirred using an Ultra-Turrax (T25 digital, Staufen, Germany) at 80 °C for 20 min. This step was considered “level 2” to characterize the fully branched starch (intact molecular structure). The next step was devoted as “level 1” to the evaluation of separate chain branches of starch. In this phase, the 3D-printed objects (10 mg) were dissolved in the prepared DMSO/lithium bromide dispersion. After that, it was debranched using the enzyme isoamylase in an acetate buffer (pH 3.5).^19^ The dispersions of the debranched starch were neutralized *via* sodium hydroxide (0.2 M) to a pH of about 7. Then, they were freeze-dried using a freeze-dryer device (Martin Christ, Alpha 1-2 LD plus, Osterode, Germany) and finally redissolved in the DMSO/lithium bromide dispersion through a magnetic stirrer for 120 min at 80 °C.

### 2.5 Molecular Characterization by Size-Exclusion Chromatography

Size-exclusion chromatography (SEC) separates macromolecules according to their respective corresponding hydrodynamic radius (*R*_h_) or hydrodynamic volume (*V*_h_). For a complex branched polymer such as fully branched starch, there is no unique relationship between size and molar mass. The SEC weight distributions *w*(log *V*_h_) include the fully branched starch (*w*_br_(log *V*_h_)) or debranched starch (*w*_de_(log *V*_h_)). Moreover, the degree of polymerization (DP) of debranched starch (“level 1”) and the average hydrodynamic radius (*R̅*_h_) of fully branched starch (“level 2”) were determined according to the procedures set out in detail elsewhere. Then, the molecular structures of starches were monitored through an SEC device (Agilent Technologies, Waldbronn, Germany) coupled with an isocratic pump, a series of separation columns (GRAM precolumn, GRAM 30, and 3000 analytical columns, Polymer Standard Services, Mainz, Germany), and a refractive index detector (RI; Shimadzu RID-10A, Shimadzu Corp., Kyoto, Japan). The mobile phase was DMSO containing 0.5% (w/w) lithium bromide solution, which was filtered with a hydrophilic Teflon membrane filter (0.2 μm pore size and 47 μm diameter, Millipore Billerica, MA). The separation columns were kept at 80 °C, and the RI detector was set at 48 °C. The flow rate was adjusted at 0.6 mL min^–1^. A series of pullulan standards (Polymer Standard Services, Mainz, Germany) with different average molar masses ranging from 0.342 to 16,600 kDa were utilized for conventional calibration. The specific RI increment value, d*n*/d*c*, for a definite linear polysaccharide was obtained to be identical to that of amylose, 0.0689 mL g^–1^, as they are debranched. Each pullulan standard (2 mg) was dissolved in the DMSO/lithium bromide solution (2 mL) with a magnetic stirrer for 150 min at 80 °C. The Mark–Houwink parameters for this eluent at 80 °C are *K* = 2.424 × 10^–4^ d L g^–1^ and α = 0.68.^19^ For convenience, the results of hydrodynamic volume (*V*_h_) are offered based on the assigning hydrodynamic radius (*R*_h_) as follows
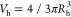
1

Each SEC chromatogram was evaluated by PSS WINGPC Unity Software (PSS Polymer Standards Service GmbH, Mainz, Germany), normalizing to obtain a similar AP peak height. The detector signal provides the size distribution values (*w*(log *V*_h_)), which were plotted against log *R*_h_.^19^ This offers the size distribution as a function of *V*_h_ or *R*_h_, which is a molecular quantity independent of the SEC set-up. This allows the resulting data to be reproduced, while showing such data as elugrams in terms of volume or elution time cannot since the elution differs with the specific SEC setup.

### 2.6 Dynamic Viscoelasticity Measurement

To accomplish dynamic analyses of viscoelastic systems, a stress-controlled rheometer, AR 2000ex rheometer (TA Instruments, New Castle, DE), equipped with a 2, 20 mm diameter cone plate at a controlled temperature of 37 ± 0.5 °C was used. The starch samples (exactly 2 g) were transferred to the cone plate and softly packed to eliminate air. Two dynamic assays were carried out: (a) an oscillatory strain sweep analysis was made to set the upper limit of the linear viscoelastic region (LVR) in the range of 0.01–100% with a constant frequency of 1 Hz at 37 °C. (b) A frequency sweep test over a range of 0.1–100 Hz at 37 °C was performed within the oscillatory strain sweep within LVR. Viscoelastic parameters, elastic or storage modulus (*G*′, Pa), and viscous or loss modulus (*G*″, Pa) as a function of angular frequency (ω, Hz) were investigated.^20,21^

### 2.7 X-ray Diffraction

To monitor the crystalline structure of starch samples as affected by DIW 3D printing, X-ray analytical instrumentation (TTR-III, Rigaku, Japan) was used. Initially, starch samples were conditioned in a desiccator containing a saturated NaCl solution (relative humidity of 75.1%) at 25 °C for 36 h to reach a moisture content of 13.6 g/100 g. The samples were exposed to the X-ray beam at 200 mA and 40 kV. The scanning region of the diffraction angle (2θ) was from 3 to 45° with a step size of 0.03°. To measure relative crystallinity degree (RCD), the total area (*I*_t_) and the area under X-ray diffraction (XRD) peaks (*I*_p_) were obtained *via* the software supplied by the manufacturer (EVA, Version 9.0), and the RCD was determined by the following equation
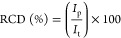
2

### 2.8 Small-Angle X-ray Scattering

Each 3D-printed starch sample was dispersed into distilled water to form a slurry (40% w/w) and slightly stirred with 56 s^–1^ (367 G-force) at ambient conditions overnight. Next, it was centrifuged (Eppendorf centrifuge 5417R, Hamburg, Germany) at 5000 G-force for 5 min, and the wet precipitate was collected. A Bruker small-angle X-ray scattering (SAXS) instrument (NanoSTAR, Bruker AXS Inc., Billerica, MA) was used to record the two-dimensional (2D) scattering pattern of printed starches at 50 kV and a 30 W Cu Kα radiation wavelength of 1.5418 Å. The SAXS instrument was equipped with a Vantec 2000 detector and pinhole collimation for point focus geometry. The one-dimensional (1D) scattering curves were obtained in the range of 0.2 < *q* < 1.4 nm^–1^ from the 2D scattering patterns through the built-in software. The SAXS curves were further analyzed with the help of the 1D linear correlation function *L*(*r*) (eq 3)
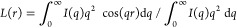
3Here, *q* is the scattering vector, *I*(*q*) is the scattering intensity, *r* is the distance in real space, and the denominator is the scattering invariant.

### 2.9 *In Vitro* Digestion

For *in vitro* digestion, 100 mg of starch (powder) was dispersed in 2 mL of distilled water and incubated at 37 °C for 5 min in a 50 mL centrifuge tube. Then 8 mL of an enzyme solution containing 0.67 mg of pancreatin and 33.3 μL of amyloglucosidase in sodium acetate buffer (0.2 M, pH 6.0) was added with moderate magnetic stirring. Volumes of 0.1 mL aliquots were then placed into 0.9 mL of absolute ethanol at 0–250 min, and the glucose concentration was determined by the d-glucose (GOPOD Format) assay. The concentration of glucose so obtained was transformed into the concentration of starch digested by multiplying by 162/180, which is the molar mass ratio between glucose and AGU (the starch monomer unit). Starch digestion curves were then plotted as the percentage of starch digested *versus* time.^7^

### 2.10 Fitting to First-Order Kinetics

The starch digestion curves were fitted to an integrated first-order equation (eq 4)
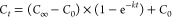
4Here, C_t_ is the percentage of starch digested at time *t* (min), C_∞_ is the percentage of starch digested by the end of reaction time, C_0_ is the starch digested at *t* = 0, and *k* is the starch digestion rate coefficient. Different digestion phases, if present, were identified by using the logarithm-of-slope (LOS) analysis method described in detail elsewhere^22^ through a transformed equation (eq 5)
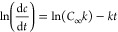
5

The values of *k* so obtained were refined by a nonlinear least-squares (NLLS) fit method to avoid the inaccuracy of numerical derivatives in the LOS method.

### 2.11 Printing Quality Assessment

Each 3D-printed object was transferred into a specific chamber of (20 × 20 × 20) cm^3^ to be photographed using a digital camera (α 7M3 E-Mount, Full-Frame Mirrorless, 24.2 MP, Sony, Tokyo, Japan).

### 2.12 Statistical analysis

All instrumental experiments were carried out in triplicate with the arithmetic mean and standard deviation of the data calculated and reported. Analysis of variance was utilized in the determination of the main effects and to examine the independence or the interactions between various factors on the instrumental and sensory data. Duncan’s multiple range test was applied to separate means of data when statistically significant differences (*p* < 0.05) were observed.

## 3 Results and Discussion

### 3.1 Characteristics of Starch Varieties

The chemical composition of starch importantly influences its functional properties, including the formation of a starch polymer network, enzyme susceptibility, capacity of water binding, and crystallinity.^23,24^ Concerning the chemical composition of starch, a substantial difference in the AM contents or total AM/AP ratios of starch cultivars was observed (Table 1). Starches in most of the cereal, pseudo-cereal, and root flours consisted of 15.4–29.3% AM, which were representative of the starches from the normal varieties of those crops.^23^ The average AM content of RW, RP, RM, HAM, and RB starches was detected to be 21.2, 16.3, 24.8, 72.4, and 27.5 (% w/w), respectively, with an AM/AP ratio of 1:4.4, 1:6.1, 1:4.2, 1:1.3, and 1:3.6, respectively (Table 1). The AM contents of the waxy starch varieties, however, were not significantly different for the cereal and tuber cultivars (2.7–3.1%) (*p* > 0.05). The obtained results revealed a notable difference in AM content between the starch varieties, where the HAM starch was the richest in AM, as expected, and the poorest AM content occurred in the RP starch. Although the obtained values were in the reported range for the starch varieties,^23^ differences related to the cultivar or origin could be detected.

**Table 1:** Physicochemical Properties and Chemical Composition of Starch Samples from Different Botanical Sources^a^

starch type	moisture (% w/w)	lipid (% w/w db)	protein (% w/w db)	AM (% w/w of starch)	AM/AP ratio	starch damage (% of starch)	WHC (% w/w)
Wheat							
RW	14.3 ± 0.2^b^	0.360 ± 0.061^e^	0.41 ± 0.01^c^	21.2 ± 0.2^c^	1:4.4	1.12 ± 0.08^b^	53.4 ± 1.2^b^
Maize							
RM	13.0 ± 0.3^a^	0.307 ± 0.022^d^	0.45 ± 0.03^d^	24.8 ± 0.6^d^	1:4.2	1.20 ± 0.09^c^	45.2 ± 1.4^a^
HAM	14.7 ± 0.4^c^	0.290 ± 0.010^d^	0.36 ± 0.02^b^	72.4 ± 1.3^f^	1:1.3	1.16 ± 0.04^b^	87.6 ± 1.5^f^
WM	12.8 ± 0.3^a^	0.082 ± 0.004^a^	0.37 ± 0.01^b^	2.7 ± 0.4^a^	1:8.4	1.97 ± 0.3^e^	82.2 ± 1.3^e^
Potato							
RP	13.9 ± 0.6^b^	0.153 ± 0.024^c^	0.31 ± 0.02^a^	16.3 ± 0.3^b^	1:6.1	0.82 ± 0.05^a^	59.3 ± 1.3^c^
WP	14.8 ± 0.3^c^	0.089 ± 0.005^a^	0.46 ± 0.04^d^	2.8 ± 0.3^a^	1:7.9	1.23 ± 0.07^c^	77.3 ± 1.4^d^
Barley							
RB	14.9 ± 0.5^c^	0.355 ± 0.033^e^	0.60 ± 0.02^e^	27.5 ± 0.5^e^	1:3.6	1.45 ± 0.11^d^	44.3 ± 0.9^a^
WB	13.3 ± 0.4^a^	0.107 ± 0.021^b^	0.40 ± 0.03^c^	3.1 ± 0.4^a^	1:7.4	1.94 ± 0.09^e^	81.4 ± 1.7^e^

a a–f means (three replicates) that within each column, different letters are significantly different (*p* < 0.05), Duncan’s test.

Table 1 also lists the value of WHC for different starches. There was a greater WHC value concerning the waxy starches compared to their related non-waxy counterparts. This was possibly associated with the supramolecular structure of the highly branched starch fraction, which could be prominently more complex structures in comparison with AM. The WHC of native starches is principally a property of AP fractions, in which AM acts as a dilutant and an inhibitor of AP swelling^25^ or the inhibitor of AP water uptake.^26^ The lowest WHC among all studied starches was related to the RM and RB starches (*p* < 0.05), and there was no significant difference between these samples in the case of WHC (*p* > 0.05). Compared to RM and RB, a higher WHC was detected regarding RP and RW starches (Table 1). It is interesting to note that the highest WHC value among both waxy and non-waxy starch varieties was detected for the HAM. It has been reported that the high-amylose starch absorbs more water at a low temperature in comparison with waxy or normal starches but swells slowly and has a low viscosity even if heated at a high temperature.^27^

Because of the intrinsic imprecisions of the Soxhlet technique associated with the low-lipid amount measurement, the lipid levels of different starches could not be precisely detected. The Soxhlet data showed that the levels of lipids in the different starches were low as reported in the literature.^23,24^ The regular cereal starches included greater lipid amounts in comparison with potato starch (Table 1). In this case, the highest quantity of lipids occurred at RW, RM, and RB starches, which can rationally develop different crystalline structures upon the DIW 3D printing process. About the regular starches, a lower lipid level was found for waxy starch samples (Table 1). There is a positive correlation between amylose and lipid contents.^28^ The plant source, endosperm physical structure, polysaccharide composition, and starch isolation technique justify the difference in the ratio of minor components.^29^ Compared to the waxy starches, the regular starches also showed a lower level of damage starches, denoting an inferior resistance of the non-regular starch granules to mechanical stress (Table 1). Besides, the tuber starches (*i.e.*, regular and WPs) possessed the lowest starch damage levels among all tested starch samples. A similar result was reported by Schirmer et al.^24^ The obtained distinctive property is likely related to milder extraction and refining processes compared to the isolation of cereal starches.^24^

### 3.2 Morphological Evaluation of Starch Varieties

**Figure 1:**
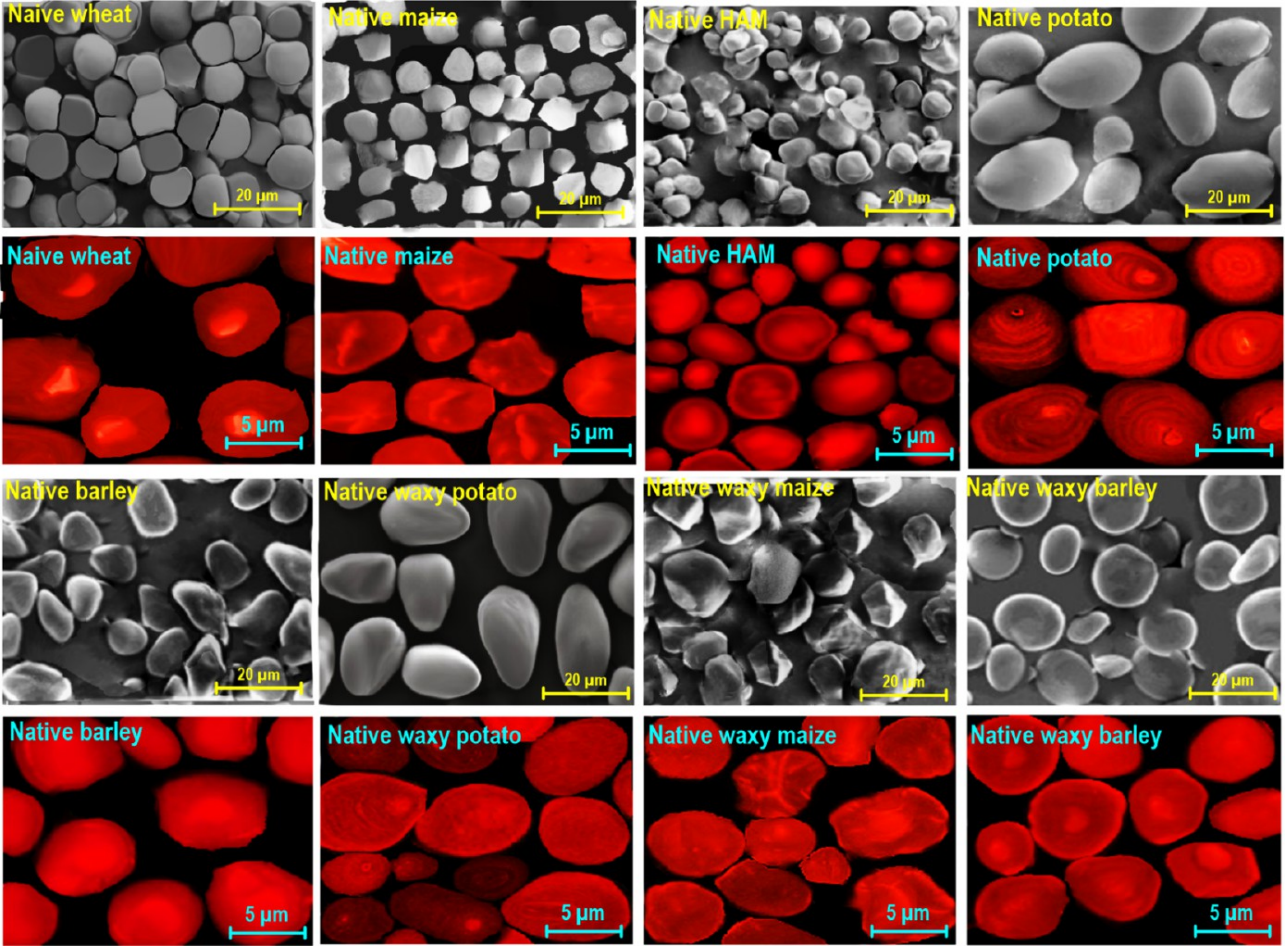
SEM and CLSM photomicrographs of different precursor grains.

The morphological properties of the original granules were explored through scanning electron microscopy (SEM) (Figure 1) (Supporting Information, Section S2). Native RW granules showed a large portion of regular spherical or ellipsoidal shapes with smooth edges and diameters in the range of 8–18 μm. The RM granules (size 4–12 μm) possessed a characteristic oval or polyhedral shape with mostly smooth, although occasionally porous, surfaces. Native HAM granules (size 2–10 μm) showed considerably heterogeneous shape (including angular, horn-like, bell-like, polygonal, and conical shapes) fractions. Native WM granules showed a polyhedral shape with rather uneven structures, possessing diameters in the range of 7–18 μm. In this case, there was no obvious difference between waxy and normal maize starches for the original granules in either their shape or the appearance of their surfaces. The native RP granules (size 12–35 μm) had oval or spherical shapes, with relatively polydispersed sizes and smooth surfaces. Compared to RP, the shell structures detected on the surface of granules WP (with diameters ranging from 9 to 26 μm) were found to be thinner and smoother. This shows a more even shell structure with a more ordered shell packing in the granule for WP in comparison with RP. Native RB granules (size 2–14 μm) consisted of a mixture of large and small granules, which were rather smooth, and had an elliptical-shaped morphology. Furthermore, some granules had small visible cracks in the central parts. The native WB granules (diameter ranging from 5 to 18 μm) were a mixture of spherical/disk/lenticular shapes. Moreover, these granules, particularly the bigger ones, had moderately small depressions.

Confocal laser scanning microscopy (CLSM) can be used as a versatile technique to explore the internal and cross-sectional structures of starch granules without disorganizing the microstructural properties of starch. The specific dye of aminofluorophore, 8-amino-1,3,6-pyrenetrisulfonic acid, was utilized to react with the reducing ends of AM and AP in the starch granules (Supporting Information, Section S3). In the present work, CLSM could detect the internal channels, growth rings, and central amorphous areas of granules (Figure 1). Compared to AP, a greater molar proportion and smaller sizes of the reduced ends per glucose residues were detected in AM. The CLSM photomicrographs exposed the presence of large A-type granules of RW, exhibiting uniformly spherical or disk-like shapes. Compared to other starch types, the internal channels were more observable in RM or WM cases. The HAM presented greater fluorescence intensity in the center (hilum) of the granules in comparison to both RM and WM. The occurrence of higher AM levels in the hilum region could justify the higher fluorescence intensity of the hilum. Compared to WP, the growth rings and the hilum in RP appeared much sharper with a greater fluorescence signal. This could be due to the greater AM level for RP. It is interesting to note that the RB consisted of regular large granules, having elliptical and lenticular A-type shapes. The B-type WB appeared to be comparatively smaller and more spherical when compared to RB.

### 3.3 Starch Molecular Characterizations

**Figure 2:**
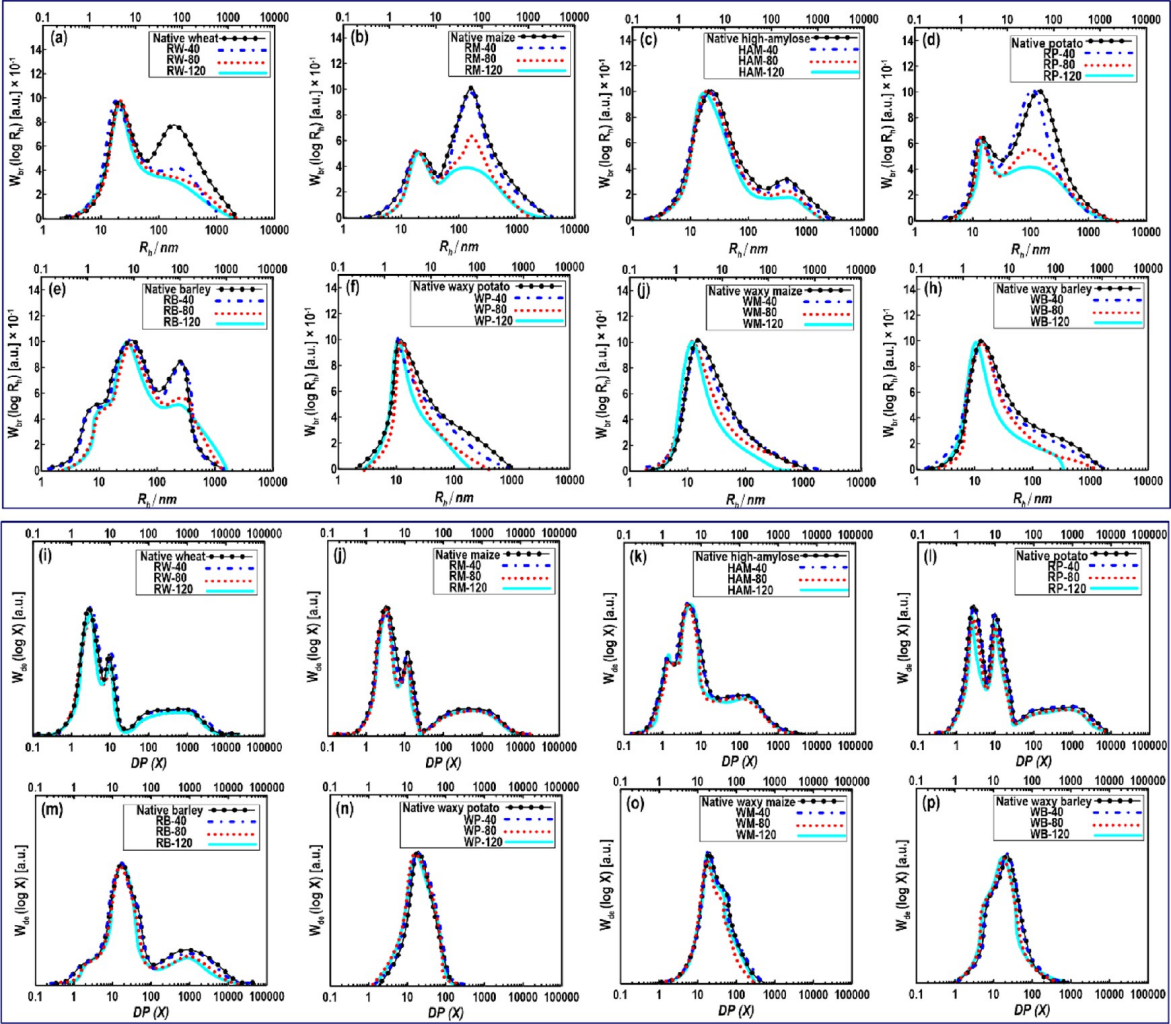
(a–h) Weight molecular size distributions of fully branched starch, *w*_br_(log *R*_h_), normalized to yield the same height as the highest peak. (i–p) SEC weight chain-length distributions, *w*_de_(log *R*_h_), of debranched starches normalized to yield the same height as the highest peak.

SEC is employed to evaluate the macromolecular and conformational properties of polymers. It separates polymers by molecular size, specifically the *R*_h_, in which there is no unique relation between size and molar mass regarding a complex branched polymer or polymer mixture of different structures (*i.e.*, fully branched starch).^7,30−32^ However, a unique relation between *R*_h_ and molar mass (or alternatively the DP, X) exists concerning a linear polymer fraction such as debranched starch.^7,33^ The SEC separation of the whole AP compound suffers from unavoidable shear-induced molecular degradation and calibration problems, so this component of *w*_br_(log *R*_h_) is not considered further.^19^ The AM component of the whole molecule distributions was expressed as the value of *R*_h_ at the peak and the average *R*_h_ (*R®*_h_) of this component.^19^Figure 2a–h shows the SEC weight molecular size distributions, *w*_br_(log *R*_h_), of whole (fully branched) starch varieties treated at different temperatures of the DIW 3D printing. All the non-printed (native) regular starches (Figure 2a–d), excluding RB, showed a typical bimodal distribution of fully branched starch, consisting of AM with an *R*_h_ of lower than 100 nm and AP with an *R*_h_ of larger 100 nm peaks. Regarding RB (Figure 2e), the peak in the range of >15 nm could be associated with the protein residues caused by the partial protein hydrolysis before starch extraction.^34^ The whole-molecule distribution of waxy starch varieties displayed an AP peak with no obvious AM peak (Figure 2f–h).

As shown in Figure 2, it is obvious that the high molecular size and extremely branched primary structure of AP were preferentially affected by degradation upon the DIW 3D printing process, especially after “moderate” and “extreme” processes. Furthermore, the *R*_h_ of AP peak for 3D-printed starches was moved to a lower distribution compared to corresponding untreated counterparts. This proposes the degradation of starch chains upon processing with the extrusion 3D printer (Figure 2f–h). There is random cleavage of the glycosidic linkages in the branches of AP upon mechanical or shearing force induced by DIW 3D printing with a more noticeable effect adjacent to the rigid crystallites in the granular starches.^7,17^ The SEC weight molecular size distributions also showed that the amylose-rich samples (like RM, HAM, and RB starches) were less affected by the DIW 3D printing process compared to the wheat and potato starches. Meanwhile, the peak of whole-molecule distribution regarding the waxy starches was decreased more by the 3D printing process. It should be noted that there was no significant difference among the *R*_h_ positions of the AM peak of 3D-printed starches. It has been reported that the AM molecule is adequately small to be stable against shear degradation under this DIW 3D printing condition.^7^ Therefore, the observations suggest that starches with comparably high AP content were more sensitive to the applied temperatures upon extrusion-based 3D printing than the amylose-rich starches.

The result of the average *R*_h_ (*R®*_h_), obtained by the SEC weight distributions of the fully branched starch molecules, also confirmed the specific molecular degradation upon application of higher temperatures during the 3D printing process (Table 2). In this case, the average *R*_h_ of 3D-printed starches was lower in comparison with untreated starch, where the *R̅*_h_ decreased more for the samples printed with “moderate” and “extreme” printing processes. In this case, the degradation of starch polymeric chains was more distinct as the temperature was applied beyond 80 °C (*i.e.*, extreme conditions). This represents that the elevated temperature causes greater damage to the polymeric structure, which was also consistent with the weight molecular size distributions of fully branched starch, *w*_br_(log *R*_h_), the result obtained from the previous research study.^7^ It was also reported that AP is more susceptible to shear degradation than the AM component owing to its comparatively inflexible structure and great molecular size.^17^ Thus, the *R®*_h_ and *R*_h_ reduction of AP could have some contribution from the degradation of a small number of AP chains.

**Table 2:** Molecular and Structural Parameters of 3D-Printed Starches Prepared at Different Processing Conditions^a^

		*R*_h_ (nm) at peak			
starch type	*R®*_h_ (nm)	AP peak	AM peak	crystallinity (%)	V-type crystallinity (%)
RW	86 ± 2^c^	23 ± 2^c^	202 ± 6^c^	43 ± 1^c^	2
RW-40	71 ± 1^c^	19 ± 1^c^	193 ± 6^c^	43 ± 2^c^	2
RW-80	55 ± 2^b^	14 ± 1^b^	163 ± 5^b^	32 ± 1^b^	8
RW-120	36 ± 1^a^	14 ± 1^a^	132 ± 4^a^	22 ± 1^a^	8
RM	77 ± 3^c^	23 ± 1^c^	172 ± 4^d^	44 ± 1^c^	trace
RM-40	73 ± 3^d^	19 ± 1^d^	175 ± 5^e^	45 ± 1^d^	2
RM-80	41 ± 2^e^	12 ± 1^e^	122 ± 3^f^	47 ± 1^e^	5
RM-120	42 ± 1^c^	11 ± 1^c^	102 ± 3^c^	43 + 1^c^	5
HAM	99 ± 4^c^	23 ± 2^c^	383 ± 7^c^	43 + 1^c^	2
HAM-40	96 ± 2^b^	23 ± 1^b^	386 ± 5^b^	43 + 2^c^	trace
HAM-80	63 ± 2^a^	18 ± 1^a^	286 ± 6^a^	43 + 1^c^	2
HAM-120	56 ± 3^c^	17 ± 1^c^	257 ± 6^d^	32 + 1^b^	5
WM	14 ± 1^d^	17 ± 1^d^	nd**	22 + 1^a^	nd
WM-40	14 ± 1^e^	17 ± 1^e^	Nd	44 ± 1^c^	1
WM-80	8 ± 1^c^	12 ± 1^c^	Nd	45 ± 1^d^	trace
WM-120	7 ± 1^c^	10 ± 1^c^	Nd	47 ± 1^e^	2
RB	67 ± 2^b^	18 ± 1^b^	158 ± 3^b^	43 + 1^c^	trace
RP-40	63 ± 3^a^	15 ± 1^a^	107 ± 5^a^	nd	nd
RP-80	43 ± 3^c^	10 ± 1^c^	74 ± 3^d^	53 ± 2^c^	nd
RP-120	19 ± 2^d^	8 ± 1^d^	53 ± 2^e^	43 ± 1^a^	nd
WP	14 ± 1^e^	25 ± 1^e^	Nd	43 ± 1^a^	1
WP-40	12 ± 1^c^	21 ± 1^c^	Nd	49 ± 1^b^	trace
WP-80	10 ± 1^c^	13 ± 1^c^	Nd	56 ± 1^d^	nd
WP-120	7 ± 1^b^	9 ± 1^b^	Nd	nd**	trace
RB	89 ± 3^a^	30 ± 1^a^	195 ± 7^a^	Nd	3
RB-40	93 ± 4^c^	28 ± 1^c^	196 ± 8^d^	53 ± 2^c^	5
RB-80	14 ± 1^d^	19 ± 1^d^	Nd	43 ± 1^a^	8
RB-120	10 ± 1^e^	14 ± 1^e^	Nd	43 ± 1^a^	8
WB	10 ± 1^c^	15 ± 1^c^	Nd	49 ± 1^b^	trace
WB-40	8 ± 1^c^	12 ± 1^c^	Nd	56 ± 1^d^	nd
WB-80	8 ± 1^b^	11 ± 1^b^	Nd	nd**	nd
WB-120	6 ± 1^a^	8 ± 1^a^	Nd	Nd	nd

a a–f means (three replicates) that within each column, different letters are significantly different (*p* < 0.05), Duncan’s test. The estimate of the *R*_h_ at the middle of a shoulder was obtained by using the first derivative of the SEC weight distribution at the point nearest to zero. ** Not detected.

The SEC weight distributions of the debranched starch samples, *w*_de_(log *R*_h_), are presented in Figure 2i–p. The regular starches (Figure 2i–m) show the normal qualitative presence of a bimodal component of AP (DP ≲ 100) and a long-chain portion associated with the branches of AM (DP ≳ 100) as reported in numerous examples in the literature.^7,17,19^ The SEC weight distributions of waxy starches showed only molecules of the AP fraction with a peak DP ranging between 1 and 1200 nm (Figure 2n–p). The chain-length distributions of the debranched native and 3D-printed starches illustrated a little difference throughout the whole distribution. This is not surprising as AP has a great number of chains, where the extrusion shearing force breaks the molecules into moderately smaller fragments with the rupture of a slight portion of individual bonds.^7,35^ To the best of our knowledge, there is no study in the literature dealing with the effect of DIW 3D printing on the molecular properties of starches to better compare our results with previously published papers. However, we recently showed that the peak of the AP branch chain fraction was unchanged in the debranched maize starch after the DIW 3D printing process.^7^ Some authors stated the impact of typical extrusion cooking on the molecular state of starch. Zhang et al.^36^ reported the lack of a qualitative difference in chain-length distributions of debranched starch products between native and 3D-printed HAM. Li et al.^17^ also found that the extrusion process did not change AP branch chain-length distributions of maize starch with different AM contents.

### 3.4 Viscoelastic Evaluation

**Figure 3:**
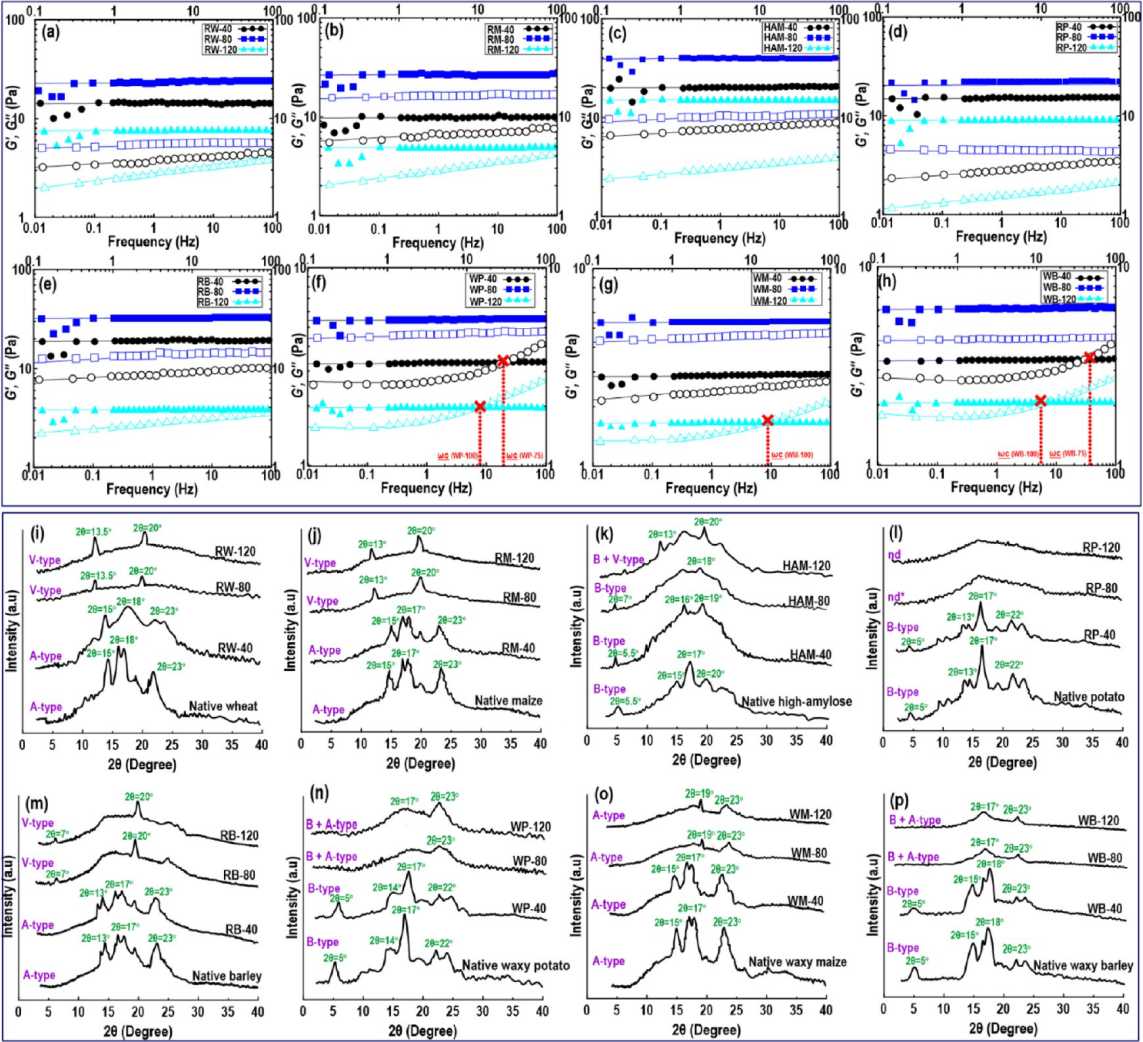
(a–h) Changes in the dynamic moduli of starch varieties as a function of frequency, where elastic modulus, *G*′, is indicated by solid symbols and loss modulus, *G*″, is indicated by open symbols. (i–p) XRD patterns of starch varieties processed with different printer temperatures of extrusion 3D printing systems. The phrase “nd” in RP-80 and RP-120 indicates that the crystalline pattern was not detected.

The frequency dependence of the elastic (*G*′) and viscous (*G*″) moduli was determined through dynamic frequency sweep experiments in the limit of the LVR (Figure 3). The normal starch varieties (RW, RP, RB, and RM) or HAM starch presented a typical gel-type mechanical spectrum as the *G*′(ω) values continuously prevail over the *G*″(ω) curves along with the whole frequency sweep test (Figure 3a–e). Irrespective of starch type, the “moderate” condition (80 °C) of 3D printing led to an increase in both *G*′(ω) and *G*″(ω) moduli in comparison with “mild” and “extreme” conditions. In the “mild” printing condition (40 °C), the starch still upheld its granular structure in the system as an intact and unbroken matrix. However, the collision between swollen granules and the motion of starch polymer chains would both be enhanced as the temperature rises in the “moderate” condition. A further increase in the printer temperature to the “extreme” condition caused a decline in both viscoelastic moduli. This can be owing to the greater extent of molecular degradation of the starch polymers as a result of advanced shearing force and temperature.^7,37,38^ Concerning the regular starch types (RW, RP, RB, and RM), the *G*′(ω) and *G*″(ω) moduli of tuber starch were lower than those of the cereal starches but still retained the dominance of elastic feature throughout the frequency measurement. The dynamic rheological data also revealed that HAM and RB starches presented the highest viscoelastic moduli among all evaluated starch varieties. In these cases, their *G*′(ω) values were comparatively independent of the frequency (0.05 < slope < 0.1), although the *G*″(ω) values were appreciably dependent on the frequency with always *G*′(ω) > *G*″(ω) (0.1 < slope < 0.3). This type of spectrum is occasionally related to weak-gel behavior.^39^ Based on their mechanical spectra, gels can be usually classified into two major categories, strong and weak gels.^40^ Strong gels have the characteristics of true gels where under small deformation conditions, they manifest typical behavior of viscoelastic solids, and above a critical deformation value, they rupture rather than flow. Weak gels, on the other hand, possess intermediate rheological properties between solutions and strong gels. Under small deformation, weak gels resemble strong gels in their mechanical behavior, but as deformation increases, the 3D networks undergo a progressive breakdown into smaller clusters.

With regard to the 3D printer temperature, increasing the temperature to 80 °C (“moderate” condition) induces granule–granule collision and the collision between the swollen starch granules. Following the extrusion printing at the temperature of 120 °C (“extreme” condition), *G*′(ω) and *G*″(ω) moduli of starch notably decreased. It was shown that the macromolecules are susceptible to thermomechanical depolymerization while printing, causing the loss of their elastic gel-like character.^7^ At higher temperatures, the mobility of starch chains is increased, making the chain interactions less efficient and accordingly resulting in weakened strength.

About the waxy starches, the mechanical spectra showed that *G*′(ω) > *G*″(ω) for a small range of frequency sweep (<10 Hz), revealing an elastic or solid-like behavior of the system in this range. In this respect, the *G*′(ω) values of 3D-printed waxy samples were much lower than those detected in normal starch varieties (RW, RP, RB, and RM) or HAM starch. This could be due to a lower level of the AM fraction in waxy starches. Case et al.^41^ reported that there is a positive correlation between the elastic modulus and the AM content of the starch, which agrees with the obtained results. The *G*′(ω) of the waxy samples also decreased as the printing temperature increased. In the low-frequency region (0.01 < ω < 1 Hz), the *G*′(ω) showed almost no dependence on the frequency (0.05 < slope < 0.1), and *G*″(ω) exhibited a slight dependent on the frequency (0.1 < slope < 0.2). However, an important crossover (ω_c_) was noted between *G*′(ω) and *G*″(ω) curves at the higher frequencies (>10 Hz). The fact that the *G*″(ω) values notably increased is a good sign of a breakdown of any stress-supporting structure/network in the system. In this scenario, a maximum in *G*″(ω) indicates the point at which an adequate extent of such a stress-supporting structure is degraded to transform the gel-like system into a viscous fluid. Moreover, as the 3D printing temperature was increased to 120 °C, a huge shift in the crossover point to the lower frequency was also detected. At the low-frequency rate, the intermolecular interactions are in dynamic equilibrium and the network acts as a viscoelastic solid. As the frequency rate increases, the degradation of structure occurs more quickly compared to its reformation rate (*i.e.*, the elastic element reduces) and the system displays increased viscous fluid behavior.

### 3.5 Crystalline Properties

The XRD patterns of 3D-printed starches were collected to gain further information on the possible rearrangement changes as a consequence of DIW 3D printing. Figure 3i shows the XRD patterns of RW with main diffraction peaks at round 15, 17, 18, 20, and 23° (2θ) showing a typical A-type crystalline structure with an RCD of 45.2%. Following DIW 3D printing, the intensities of starch diffraction peaks were detected to be considerably reduced after “moderate” and “extreme” conditions. However, these printing conditions led to intensifying the V-pattern crystal structure, with strong diffraction peaks at 2θ = 13.5 and 20.0°. The diffraction peak at around 2θ = 20° is denoted by a V-pattern polymorph. The wheat starch included the highest level of lipid (Table 1), which could reasonably develop amylose–lipid complex.^42^ Therefore, RW-80 and RW-120 exhibited a V-type crystalline structure.^43^ As can be seen in Figure 3i, the V-pattern crystalline structure increased more as the extrusion temperature was increased to 120 °C. The higher temperature combined with shear stress during the DIW 3D printing process could lead to partial molecular degradation of the starch polymers, which intensified the formation of single-helix amylose–lipid complex.

The X-ray diffractogram of RM (Figure 3j) showed a characteristic A-type crystalline packing arrangement inside the starch granules with typical reflections around 2θ = 15°, 2θ = 17°, 2θ = 20°, and 2θ = 23° (RCD = 43%). The “mild” printing process induces no important changes in the XRD characteristic peaks of native maize starch.^44,45^ Similar to the wheat starch, a V-type crystallinity was formed after “moderate” and “extreme” printing processes with strong reflections at around 2θ = 13 and 20.0°. This could be due to the creation of amylose–lipid complexes following the higher temperature of 3D printing, which could hinder the AM rearrangements (retrogradation), as previously reported.^7^ This suggests that 3D-printed maize starches presented slower short-term retrogradation. A higher amount of lipids was detected in normal maize starch (Table 1), which could help to develop single-helix amylose–lipid complex upon DIW 3D printing.

The HAM (Figure 3k) shows the presence of a characteristic B-type crystalline structure with a fingerprint reflection around 2θ = 5.5° and the main peak located at 2θ = 17° (RCD = 54%). The XRD diffractogram also displayed other peaks around 2θ = 15° and 2θ = 23°. The reflection around 2θ = 20° is possibly associated with an amylose–lipid complex.^46^ However, a V-type crystalline structure does not always indicate the presence of fatty acid inside the single-amylose helix. However, a similar combination of B- and V-type polymorphs in HAM was reported before by Sievert et al.^47^ and Shamai et al.^48^ The DIW 3D printing with a “mild” condition (a temperature of 40 °C) mitigated a slight decrease of characteristic peaks, offering a rather lower crystallinity degree than the native HAM (RCD = 39%). However, the “moderate” 3D printing conditions caused a nearly fully gelatinized starch as indicated by the loss of Bragg peaks. Surprisingly, the HAM printed with “extreme” conditions (*i.e.*, HAM-120) exhibits not only some of the reflections from the B-pattern crystallinity preserved upon 3D printing but also new peaks occurring around 2θ = 7°, 2θ = 13°, and 2θ = 20°, which are associated with AM single helices organized in a V-type crystallinity type.

The diffractogram of RP as influenced by different DIW printing conditions is shown in Figure 3l. A characteristic B-type crystalline pattern was detected in native potato starch, including major diffraction peaks around 2θ = 5°, 2θ = 10°, 2θ = 13°, 2θ = 17°, 2θ = 22°, and 2θ = 24° with an RCD of 41%.^49^ The weak B-type diffraction as the original crystallites partially retained in the starch printed with “mild” conditions. As the printer temperature increased to the “moderate” and “extreme” condition, the diffraction peak intensity of native potato starch disappeared. According to this, the printer temperature governs the crystalline structure of 3D-printed potato starches, in which an “extreme” temperature entirely gelatinized starch upon the DIW printing process.

The RB shows a typical A-type crystalline with characteristic reflections at 2θ = 13, 15, 17, 18, 19, and 23° (Figure 3m), and the result was similar to the previously reported study.^50^ The diffractogram of RB printed with a “mild” condition (RB-40) was similar to that of RB with minor differences. In contrast, “moderate” and “extreme” printing processes (*i.e.*, RB-80 and RB-120, respectively) led to the complete disappearance of the typical reflection of barley, signifying that the DIW printing had typically demolished the initial crystalline structure of barley. Interestingly, the intensity of peaks around 2θ = 7° and 2θ = 20° for RB-80 and RB-120 increased, denoting the development of a V-pattern crystalline structure. This is related to the presence of fatty acid within the single-AM helix. It seems that the formation of AM single helices is favored during 3D printing with higher temperature conditions.

Compared with the normal (non-waxy) starch, the intensity of diffraction peaks near 2θ = 20° as a sign of V-pattern polymorphism is weaker for the corresponding waxy types (Figure 3n–p). This confirms that the low level of lipids in the waxy starch (Table 1) inhibited the development of V-pattern crystal complexes. In this case, WP starch showed a typical B-type pattern with diffraction intensities around 2θ = 5, 14, 17, 19, 22, and 24° (Figure 3n). The “moderate” and “extreme” printing conditions led to a significant change in the X-ray diffractograms of WP, whose X-ray pattern from a B-type crystalline structure was changed to a B- + A-type pattern. This is verified by the appearance of a peak around 2θ = 23° as a typical A-polymorph but also a clear reduction of the intensities of XRD reflections. There was a pair of double helices in the “A” and “B” arrangements. The rearrangement of a pair of double helices could lead to the transformation of crystallites.^51^

Concerning WM, a characteristic A-type crystalline structure was observed, including characteristic reflections around 2θ = 15 and 23°, along with a dual adjacent shoulder peak near 2θ = 17 and 18° (Figure 3o). As expected, the intensity of the diffraction peaks at 15° showed a decreasing trend until the peak disappeared as the 3D printing temperature was increased. A diffraction peak around 19° appeared on the diffractogram of WM-80 and WM-120 samples, specifying that “moderate” and “extreme” printing conditions could increase the short-range molecular order in the amorphous region and develop a more perfect crystalline structure.^52^

WB starch showed a characteristic B-type crystalline structure with the diffraction peaks at 2θ = 5°, 2θ = 15°, an uncertain doublet at 17 and 18, and 23°, and a dual adjacent shoulder peak near 2θ = 22 and 24° (Figure 3p). WB starch lost most crystalline structure upon “moderate” and “extreme” printing processes, and it indicated that the ordered structure was rather disrupted. However, the peaks at 22 and 24° converged gradually and developed a new crystalline reflection around 23°, where the B-pattern polymorph was transformed to a B- + A-type crystalline structure. Some studies have reported that the B-type polymorph prefers to be converted into C-type (A- + B-type polymorphs) crystalline packing under an appropriate shearing process.^52^

### 3.6 Small-Angle X-ray Scattering

**Figure 4:**
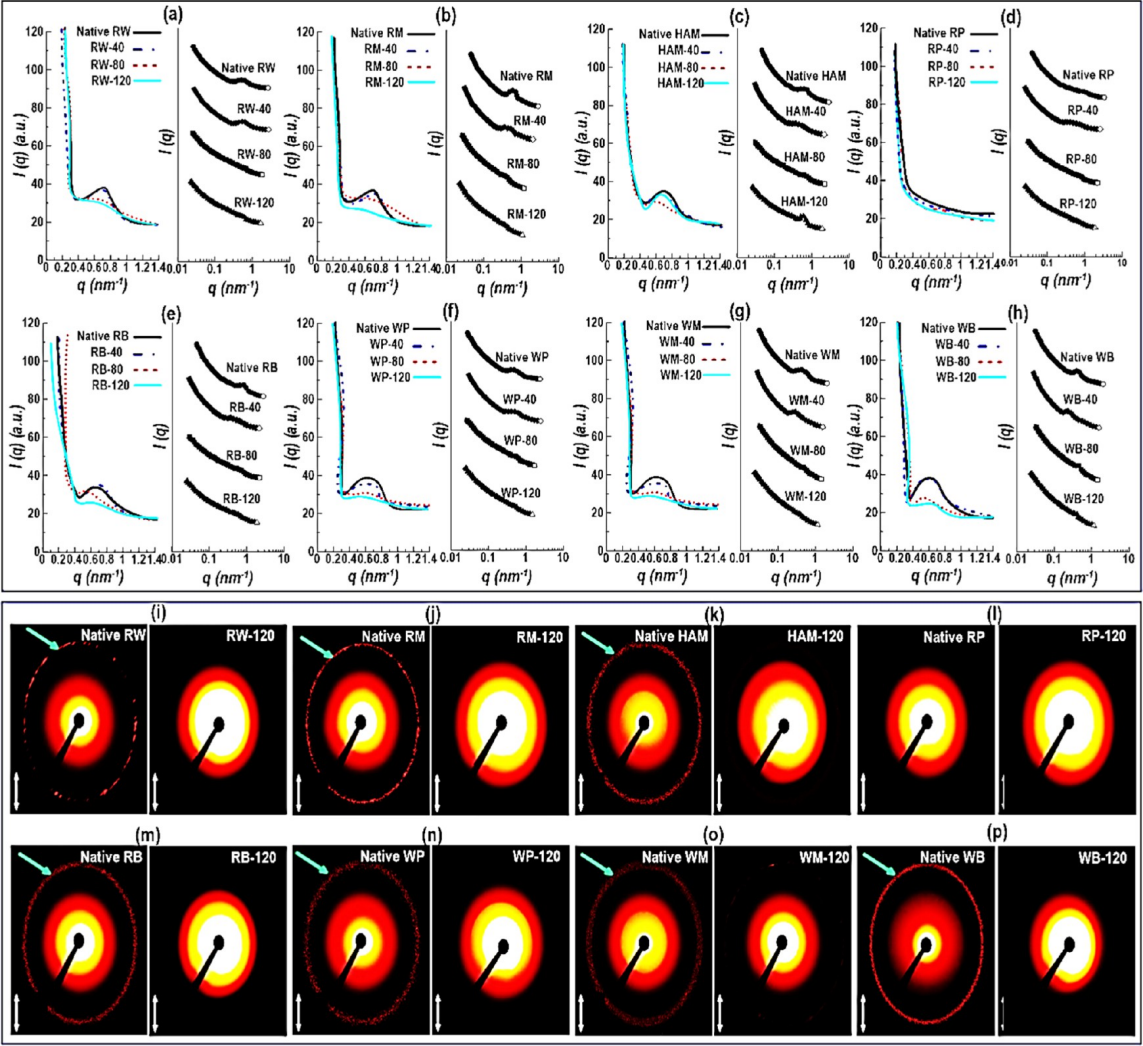
(a–h) SAXS linear (left-side) and double-logarithmic (right-side) plots; (i–p) 2D scattering images of the starch samples.

The changes in the lamellar structures of different starch varieties as affected by the DIW printing process were further explored through SAXS measurements. Figure 4a–h shows the linear and double-log scale SAXS patterns of 3D-printed starch varieties as the scattering intensity *I*(*q*) *versus* scattering wave vector *q*. At the low *q*, all the SAXS curves are characterized by an intense scattering, followed by a quick reduction at the high *q* with a typical reflection at a *q*-value of 0.6–0.7 nm^–1^. This characteristic reflection is related to the periodic arrangement of alternating amorphous and crystalline lamellae of highly branched starch polymers, which correlates well with the 9–10 nm starch semi-crystalline lamellar matrix.^53^ The only exception from presenting the interference peak was RP, whose SAXS pattern showed the lowest absolute intensity (Figure 4d). It was revealed that the lack of potato interference peak could be associated with the lower surface area per unit volume (*e.g.*, a value of ∼2600 cm^–1^ for RM, ∼900 cm^–1^ for RP), which was obtained by calculating the Porod constant at the lowest *q* values, under the limiting assumption of *q*^–4^ behavior.^54^ From Figure 4a–h, it can be seen that the scattering intensity of the lamellar peak for the waxy starches was higher than that of the normal starch varieties (RW, RP, RB, and RM) or HAM starch, indicating a higher electron density contrast (Δ*r* = ρ_c_ – ρ_a_, where ρ_c_ and ρ_a_ are the electron densities of the crystalline regions and the amorphous regions in the semi-crystalline lamellae) between crystalline and amorphous lamellae.

Concerning the effect of the 3D printing process, the native starches showed a well-defined SAXS peak, suggesting the periodic lamellar arrangement of starches in the semi-crystalline state with a repeat distance of ca. 9–10 nm (Figure 4a–h). On the contrary, extrusion 3D printing makes an important change in the lamellar and supra-lamellar size range, which was strongly dependent on the printing temperature. In the lamellar region, there is a gradual decrease in *q* intensity, consistent with a previous report.^53^ The SAXS patterns also show that the 9 nm scattering peak becomes less prominent after DIW printing with “extreme” conditions. This denotes a disruption of the lamellar structure of granular starch upon the 3D printing process with a corresponding decrease in the long-range order. The partial disruption of crystalline lamellae by DIW 3D printing could lead to the loss of the periodic lamellar structure. Temperature and shear primarily hydrolyzed the amorphous regions of the starch granule.^7^ Therefore, it was expected that there is no possibility of developing a periodic lamellar structure resulting from a significant level of glycosidic bond cleavage and breaking of the crystalline lamellae.

The obtained 2D scattering images also revealed that the 3D printing process caused a notable change in the starch lamellar structures (Figure 4i–p). In this case, even circular structures with isotropic characteristics were found for the non-printed (native) regular starches, presenting a periodic semi-crystalline structure within each native starch. As most of the crystals are randomly oriented, the scattering pattern is observed as a ring; however, the individual spots are visible because of the reflections from single large crystals. About the 3D-printed starches, there was an expansion in the bright spots located at the center of the 2D SAXS profile with an important disappearance of the expanded scattering rings in the 2D scattering patterns (Figure 4i–p). Also, the scattering pattern of regular and waxy starches was dissimilar, suggesting that there were differences in the lamellar structure of starch granules with different AM/AP ratios. In this case, the amorphous lamellae were hydrolyzed and the crystalline lamellae were partially disrupted, leading to a different periodic arrangement. However, since the diameter of bright spots is also broader, this rearrangement is not uniform. It should be noted that only the 2D scattering patterns of 3D-printed samples processed at 120 °C (“extreme”) were presented as their patterns were very similar to those of 3D-printed samples at 40 (“mild”) and 80 °C (“moderate”).

### 3.7 *In Vitro* Digestion Kinetics

**Figure 5:**
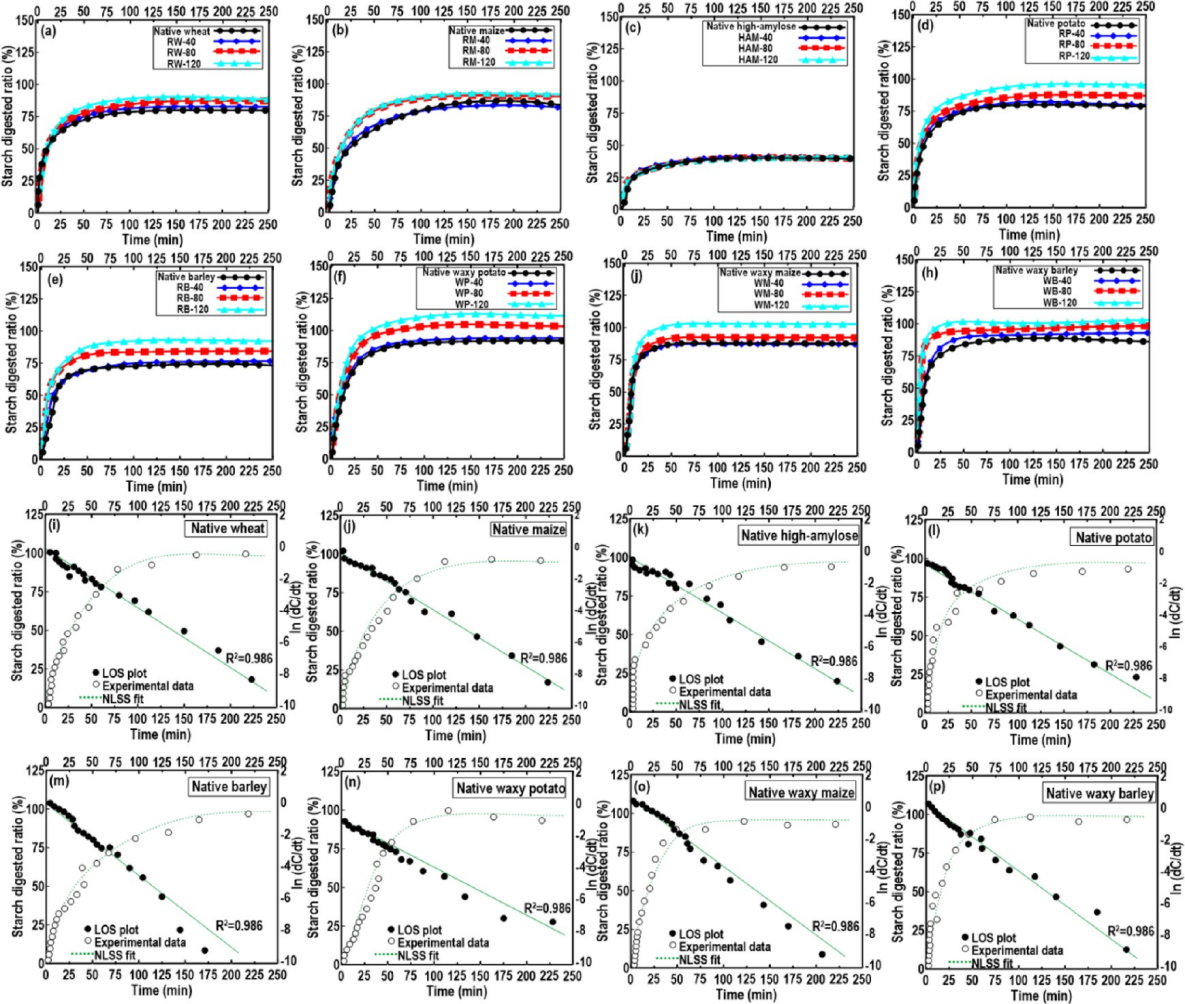
(a–h) Effect of DIW 3D printing on digestion kinetic profiles of starch varieties. (i–p) Digestion phase of 3D-printed starch samples.

There is a strong relationship between the starch molecular structure and the digestibility, in which starches with short AP branches are more susceptible to enzymatic digestion compared to those with long branch chains such as “high-amylose” starches. Figure 5a–h shows the digestion curve of different 3D-printed starches, and an example of LOS and NLLS fitting curves are shown in Figure 5i–p. As all fit trends were similar to those of native starch samples, we presented only LOS and NLLS fitting curves for the relevant native (non-waxy and waxy) starches. A characteristic first-order kinetics behavior was detected for all digestograms (Figure 5a–h), where only one digestion phase was found (Figure 5i–p). As each digestion process commenced when digestive enzymes were incorporated, the digestion process did not start at a value of 0%. There is a rapid increase in the hydrolysis rate of 3D-printed starch in the first 20 min, and following that, the digestion trend remained comparatively unchanged. During the early 20 min digestion process, a clear difference was noticed among the digestograms of 3D-printed starch varieties. In this case, each digestion curve nearly reached a plateau with about 75% starch being digested (excluding the digestion process for 3D-printed HAM), which was calculated using the fitting to the first-order kinetics equation (Table 3). Compared to 3D-printed normal (non-waxy) starches, the printed waxy varieties showed a faster hydrolysis rate with a higher degree of hydrolysis (Table 3). This may be attributed to waxy starches possessing higher enzymatic digestibility compared to normal starches, denoting an important contribution to the increase in the rapid digestion of starch.^55^

**Table 3:** Kinetic Parameters Provided by LOS Tests of *in Vitro* Digestibility Curves and Reducing Sugar Released Extent after 250 min Digestion^a^

starch type	*k* (min^–1^)	*C*_∞_	*R*^2^
RW	0.331 ± 0.033^d^	29.3 + 0.6^b^	0.985 ± 0.002
RW-40	0.338 ± 0.037^e^	28.1 + 0.8^b^	0.965 ± 0.003
RW-80	0.410 ± 0.009^f^	42.4 + 0.9^c^	0.955 ± 0.003
RW-120	0.541 ± 0.024^g^	57.3 + 0.9^d^	0.997 ± 0.001
RM	0.260 ± 0.010^d^	31.9 + 0.7^b^	0.996 ± 0.002
RM-40	0.257 ± 0.023^e^	29.2 + 0.9^b^	0.977 ± 0.001
RM-80	0.479 ± 0.025^f^	36.6 + 0.7^c^	0.967 ± 0.001
RM-120	0.588 ± 0.009^g^	48.2 + 0.7^d^	0.988 ± 0.003
HAM	0.098 ± 0.005^a^	8.41 ± 0.02^a^	0.988 ± 0.001
HAM-40	0.100 ± 0.019^d^	7.42 + 0.05^b^	0.974 ± 0.001
HAM-80	0.112 ± 0.037^e^	9.05 + 0.09^b^	0.997 ± 0.001
HAM-120	0.130 ± 0.047^f^	11.47 + 0.09^c^	0.983 ± 0.004
WM	0.465 ± 0.022^g^	42.6 + 1.2^d^	0.990 ± 0.002
WM-40	0.449 ± 0.018^h^	44.3 + 1.3^e^	0.981 ± 0.002
WM-80	0.698 ± 0.021^c^	67.1 ± 1.5^b^	0.975 ± 0.003
WM-120	0.794 ± 0.034^c^	82.7 ± 1.8^b^	0.980 ± 0.001
RP	0.333 ± 0.034^b^	28.4 ± 0.5^a^	0.987 ± 0.003
RP-40	0.325 ± 0.045^a^	27.5 ± 0.7^a^	0.976 ± 0.003
RP-80	0.424 ± 0.021^d^	52.7 + 0.8^b^	0.985 ± 0.004
RP-120	0.579 ± 0.058^e^	61.6 + 1.2^b^	0.945 ± 0.002
WP	0.379 ± 0.037^f^	38.2 + 0.5^c^	0.999 ± 0.004
WP-40	0.399 ± 0.045^g^	42.8 + 1.2^d^	0.978 ± 0.003
WP-80	0.679 ± 0.031^h^	69.7 + 1.8^e^	0.987 ± 0.002
WP-120	0.734 ± 0.054^c^	79.9 ± 1.5^b^	0.995 ± 0.001
RB	0.279 ± 0.022^c^	32.7 ± 0.8^b^	0.979 ± 0.003
RB-40	0.247 ± 0.024^b^	32.9 ± 0.9^a^	0.997 ± 0.002
RB-80	0.368 ± 0.043^a^	46.7 ± 1.3^a^	0.983 ± 0.003
RB-120	0.371 ± 0.036^d^	53.6 + 0.8^b^	0.990 ± 0.002
WB	0.389 ± 0.035^e^	37.2 + 1.0^b^	0.985 ± 0.002
WB-40	0.397 ± 0.016^f^	47.1 + 1.6^c^	0.985 ± 0.003
WB-80	0.540 ± 0.024^g^	65.8 + 1.5^d^	0.964 ± 0.002
WB-120	0.683 ± 0.039^h^	72.3 + 1.9^e^	0.987 ± 0.001

a a–f means (three replicates) that within each column, different letters are significantly different (*p* < 0.05), Duncan’s test.

To measure the first-order coefficients (*k*), LOS fitting test was used for digestion kinetic profiles. The fitted digestion rate coefficient (*k*) and percentage of total starch digested at a long time (*C*_∞_) are summarized in Table 3. A single-phase pseudo-first-order kinetic (*R*^*2*^ > 0.960) was revealed for the tested 3D-printed starches, offering a good fit to the experimental data. The digestion rate presented a considerable digestion difference, where increasing the printer temperature caused a higher digestion rate to the greatest extent (Table 3). Moreover, it can be seen that the digestion rate increased in the order of WM > WP > WB > RP > RW > RM > RB > HAM starches. This proposes a role for the AM in reducing hydrolysis rates of starches having a higher AM content and AM/AP ratio (Table 1). Among the 3D-printed starches, WM and HAM starches revealed the fastest and slowest digestion rates with the highest and lowest digestion extents, respectively. It was reported that the apparent AM content is negatively correlated with digestibility since the AM in granular starch was more resistant to digestion by enzymes.

### 3.8 Printing Performance and Microstructure Evaluation

**Figure 6:**
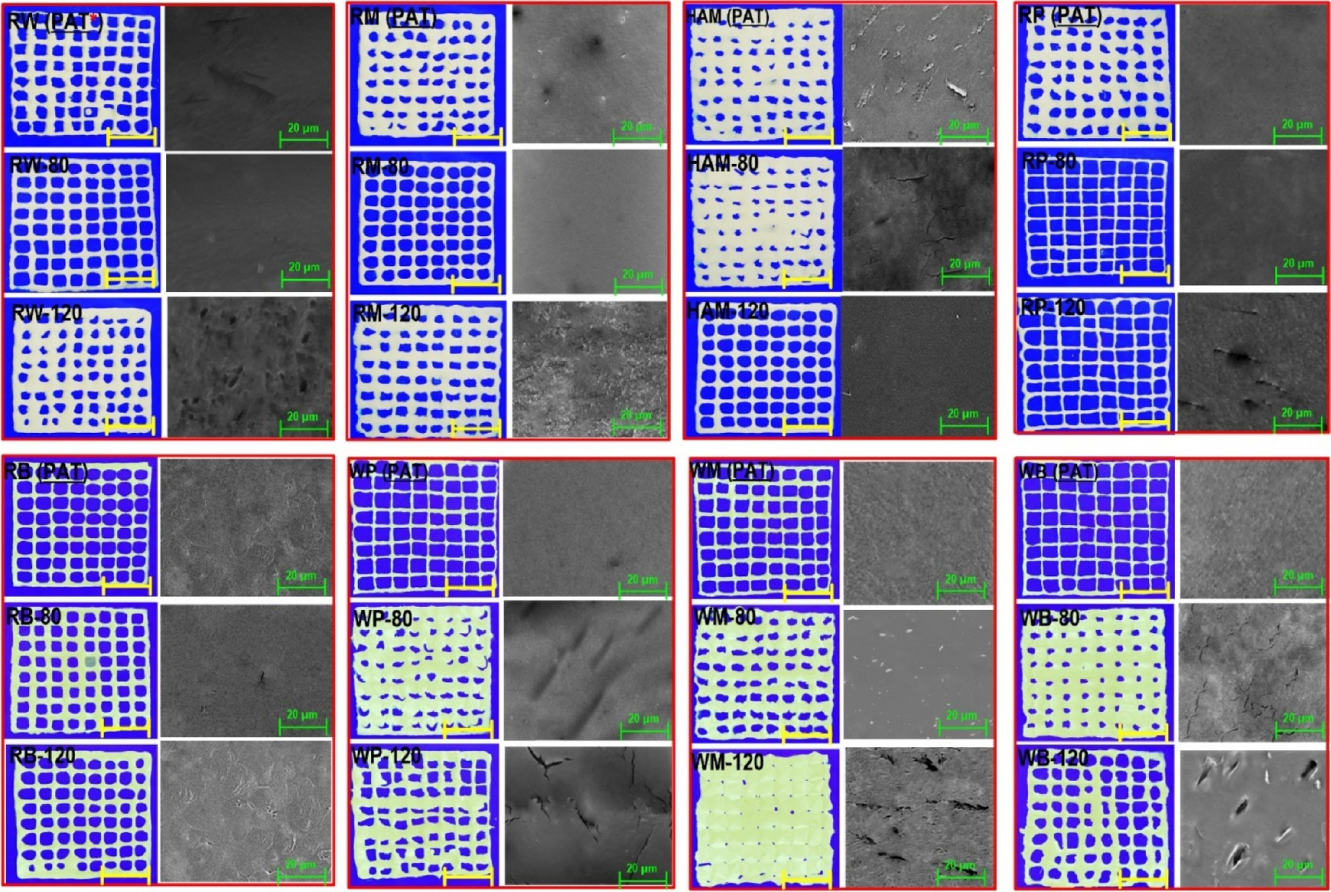
Printing performance and SEM images of different 3D-printed starches. The scale bar regarding the printing performance images is 1 cm. PAT denotes printing at ambient temperature.

To highlight the adaptability of manufactured printed starch samples, we established the competence of different starch inks to generate 3D-printed architectures. Accordingly, a geometrical shape of the lattice matrix (lattice square) was printed. The lattice square geometry resulted in a proper assessment of the printing performance and shape quality properties (edge aspect and shape reliability), simplifying the structural characterization. All starch inks were effectively extruded from the nozzle tip and manufactured into a lattice matrix (Figure 6). In this case, the starch samples (printing with no heating-induced printing temperature) were partly extruded out of the nozzle once compared to the thermally treated starch inks for which a long time was necessary (data not shown). In contrast, the 3D printing process was improved in the case of thermally treated starches. It could be seen from Figure 6 that an increase in the printer temperature up to 80 °C led to stable and formable 3D-printed starches. Once the temperature reached gelatinized temperature, the starch varieties (except high-amylose starch) can show a gel-like character with loss of their semicrystalline structure, which offers better supporting performance. This proposes that DIW 3D-printed starches (excluding high-amylose starch) treated at 80 °C could be gelatinized without part of the intact crystalline and lamellar structures retained. On the contrary, with a further increase of temperature to 120 °C, these 3D-printed objects accumulated but showed an uneven shape (Figure 6). This may be possible due to the difficulty in extrusion and the insufficient structural recovery of 3D-printed starch-based materials owing to the simultaneous application of high temperature and large amounts of shearing force. This led to the discontinuity of the line and the collapse of several layers. The printing quality results of all waxy starches showed that the printing process up to 120 °C caused a low shape fidelity, which consequently spread over the surface. This could be justified by the poor viscoelastic network and the weak gel-like structure (required to support the subsequently deposited layers) of their related inks. The printed HAM starch, in contrast, processed at a temperature of 120 °C, presented somehow better shape fidelity. It has been reported that there is a linkage between the dense packing of the biopolymers and the crystal organization inside the granular structure of HAM starch.^56^ The high temperature (<120 °C) and shearing force during extrusion processing in the presence of water could disrupt the highly ordered granular structure of the starch, which is based primarily on AP crystallites. After this disruption of the granular structure, the AM can rearrange (formation of double helical structures, crystallization, retrograde, etc.) into more thermally stable forms. In the current study, the extreme applied printer temperature was 120 °C, which is lower than the melting temperature of retrograded AM (a melting point of ∼155 °C), which is known to be enzyme-resistant.^57^ Thus, the printer conditions might promote the annealing of ordered structures (alongside conventional gelatinization) contributing to considerable resistance to enzymatic hydrolysis. In addition, it is possible that partially hydrolyzed starch polymers may have been incorporated into a dense/crystalline structure contributing toward enzyme resistance.

Among all the assessed 3D-printed starch varieties, the printed RB-80, RM-80, and RW-80 starches presented an excellent lattice square structure with a fair resolution (Figure 6). Inference of such well-defined geometries could be likely associated with a strong gel network (*i.e.*, improved *G*′), a stable molecular structure, and enhanced crystalline features of these starch varieties. In contrast, the 3D lattice square structures fabricated by all types of WM, WP, and WB starches showed low shape fidelity and poor printing performance (Figure 6). In these samples, however, their corresponding inks printed at a temperature of 80 °C showed a slightly better printing quality, which could be due to an enhancement of collision between swollen granules and adequate polymer chain interactions. This probably leads to a homogeneous gel network with increased *G*′ and accordingly higher printing performance than other waxy 3D-printed starches. It should be mentioned that the images of printing performance concerning the starches printed with “mild” conditions are not shown but presented a printing quality very close to those of the starches printed at an ambient temperature.

The SEM micrographs of 3D-printed starches, as affected by the starch type and 3D printer temperature, are schematically presented in Figure 6. The 3D-printed non-waxy starches processed with “mild” conditions, excluding the printed HAM, revealed a uniform matrix with no evidence of apparent gaps and micro-cracks on their surface. A comparable result was detected for the 3D-printed non-waxy starches processed with “moderate” conditions. Apart from the 3D-printed HAM, the “extreme” condition strongly affected the integrity and morphological structures of all the other 3D-printed non-waxy starch objects. The flat morphology of the 3D-printed non-waxy starch constructs was affected increasingly more by the higher temperatures. It was clear that some micro-cracks have appeared on the matrix, in which the surface has become much less uniform following the application of this “extreme” condition. In contrast, the surface morphology of 3D-printed HAM, processed with either “mild” or “moderate” conditions, showed a rugged and uneven morphology with agglomerated pieces detectable in the SEM micrographs. However, the surface morphology of printed HAM became uniform with a smooth morphology after the application of “extreme” conditions. Compared to the 3D-printed non-waxy starch, the morphological structure of waxy samples seemed to be more strongly affected by the DIW 3D printing process involving the utilization of “moderate” or “extreme” conditions. These presented uneven surfaces with several gaps and micro-cracks on their surfaces. This indicates that such samples could suffer from some level of degradation, in which the microstructure was damaged by the DIW 3D printing process. In contrast, the surface of 3D-printed waxy starches, when treated during the “mild” condition, offered interconnected structures with even and intact matrices.

## 4 Conclusions

The printability and molecular behavior of the 3D-printed starches based on eight different starch types with different AM/AP ratios as influenced by the temperature of an extrusion-based 3D printer were systematically investigated. The differences in the printing performances were discussed according to their molecular size, viscoelastic properties, and lamellar structures. We also evaluated a possible relationship between the degradation profile and the digestive rate of 3D starches. As was expected, AP was more susceptible to molecular degradation than AM, where a higher printer temperature promoted the molecular degradation of the AP. Upon extrusion, under both “mild” and “extreme” conditions, the 9 nm spacing was lost as revealed by the small-angle X-ray scattering experiment. The obtained data already showed that the differences regarding the molecular composition (AM contents, specific molecular parameters of both starch polymer structure fractions) of starches result in an important difference in the rheological properties and crystalline characteristics and hence a diverse 3D printing quality. It was also revealed that the printer temperature plays an important role in impacting the molecular size of starch. SEC experiments revealed that the AM fraction was more flexible toward extrusion shearing force during the application of high temperatures. This made them not easily degraded, and the AM fraction was less affected during the 3D printing process. In contrast, AP was much less flexible due to its branched structure and huge molecular size which made them more susceptible to thermo-mechanical degradation during the printing process. It has been observed that depending on the extrusion conditions used, different degrees of gelatinization are obtained. “Moderate” processing of the starches resulted in almost fully gelatinized starches, and during digestion, an A- or B-type crystallinity was developed. In contrast, “extreme” processing led to the formation of single-AM helices which resisted the digestion process and to a lower degree of gelatinization. Among the different starches and varieties, the molecular degradation was the highest in waxy starch samples (WM, WP, and WB), and also, it got degraded maximally at a comparatively lower (80 °C) temperature compared to the non-waxy varieties, which showed a maximum degradation at 120 °C. The results revealed that the AM/AP ratio and the printer temperature have a noteworthy effect on the 3D printing process. This information can be developed further and applied to optimize a printing process to fabricate better quality printed starch-based products by choosing a suitable variety of starch cultivars and optimum extrusion system parameters.

## Supporting Information Available

Analysis of chemical and physicochemical properties of samples, morphological behavior by SEM, and CLSM (PDF)
