# Supplementary material for: Insights into the Supramolecular Structure and Degradation Mechanisms of Starch from Different Botanical Sources as Affected by Extrusion-based 3D Printing
Source: Biomacromolecules. 2022 Dec 2;24(1):69–85. doi: 10.1021/acs.biomac.2c00881 (PMC9832475; doi:10.1021/acs.biomac.2c00881)
Supplement: Supplementary file 1 — bm2c00881_si_001.pdf [file bm2c00881_si_001.pdf]

“Supporting Information” for:

Insights into the supramolecular structure and degradation mechanisms of starch from different botanical sources as affected by direct-ink-write 3D printing

Mahdiyar Shahbazi[†*], Henry Jäger[†*], Rammile Ettelaie[‡], Marco Ulbrich[¥]

[†]Institute of Food Technology, University of Natural Resources and Life Sciences (BOKU), Muthgasse 18, 1190 Vienna, Austria.

[‡]Food Colloids Group, School of Food Science and Nutrition, University of Leeds, Leeds, LS2 9JT, UK.

[¥]Technische Universität Berlin, Department of Food Technology and Food Chemistry, Chair of Food Process Engineering, Office GG2, Seestraße 13, D-13353, Berlin, Germany.

S-1. Analysis of chemical and physicochemical properties of samples

The sample moisture content (%) was determined thermogravimetrically using the moisture analyzer MLB-50-3 (Kern & Sohn GmbH, Balingen-Frommern, Germany) by weight loss from the initial weight. The crude protein content of the starches and flours was determined on the basis of the Kjeldahl method using a Kjeltec™ 2400 Auto Analyzer Unit. For the conversion of the percentage nitrogen to crude protein content, the factor 6.25 was used. The crude lipid content (i.e. free lipids) was quantified using the AACC Method No. 30-25.01 (2010). The ash content was determined according to the ICC Standard Method No. 104/1 (1990) and related to the dry matter of the sample substance. The water retention capacity (WRC) was determined by measuring the water uptake of the samples (at approx. 20 °C) according to the standard AACC Method No. 56-11.02. It is expressed as percent weight of solvent retained by the sample in a gel pellet after centrifugation and decantation related to the sample weight on a 14% moisture basis. For the determination of the AM/AP content of total starch, the AM/AP assay procedure, utilizing the commercially available kit (Megazyme International Ireland Ltd.), was followed according to the recommendation of the manufacturer. This enzymatic method is based on the specific formation and precipitation of AP–Concanavalin A (Con A) complexes, after a pre-treatment of the sample to solubilize resistant starch and to remove lipids and free d-glucose. The test kit includes relative standard deviations of <5% for pure starches. The total starch content of the samples was measured enzymatically using an assay kit according to the standard AACC Method No. 76.13. The starch content measurement includes the major components amylose and amylopectin, the minor components of starch (protein, minerals, and lipids) are not detected. The damaged starch content of the samples was determined using an assay kit in accordance with the AACC Method No. 76-31.01 (2010). The method is based on the enzymatic susceptibility of damaged starch granules. Each measurement was performed in duplicate.

S-2. Morphological behavior by SEM

The morphological structure of the starch granules was examined through a scanning electron microscope (SEM, Hitachi, S-2830N, Japan). Initially, a tiny amount of freeze-dried samples was sputter-coated with a thin layer of gold/palladium at 20 mA for 4 min (JEOL JFC-1600, Auto Fine Coater, Tokyo, Japan). An acceleration voltage of 20 kV was used to avoid the samples from being damaged with a magnification objective of 3 kX.

S-3. Confocal Laser Scanning Microscopy (CLSM)

The native starch granules were stained with 8-amino-1,3,6-pyrenetrisulfonic acid (APTS) dye and visualized under a confocal laser scanning microscope (Zeiss LSM 710, Carl Zeiss MicroImaging GmbH, Jena, Germany) equipped with a 40 ×/1.3 oil objective lens. Images of optical sections of starch granules were recorded and examined with ZEN 2009 Light Edition software (Carl Zeiss MicroImaging GmbH, Jena, Germany).
